# Circulating Endothelial-Derived Apoptotic Microparticles in the Patients with Ischemic Symptomatic Chronic Heart Failure: Relevance of Pro-Inflammatory Activation and Outcomes

**Published:** 2014-09-01

**Authors:** Alexander E. Berezin, Alexander A. Kremzer, Tatayna A. Samura, Yulia V. Martovitskaya

**Affiliations:** 1 State Medical University, Internal Medicine Department, Zaporozhye, Ukraine; 2 State Medical University, Clinical Pharmacology Department, Zaporozhye, Ukraine; 3 State Medical University, Pathology Department, Zaporozhye, Ukraine

**Keywords:** Microparticles, Inflammation, Heart Failure, Survival, Prognosis

## Abstract

**Background:**

Endothelial-derived apoptotic microparticles (EMPs) play a pivotal role in endothelial dysfunction in hronic Heart Failure (CHF).

**Objectives:**

The present study aimed to evaluate the association between EMPs and pro-inflammatory biomarkers, clinical status, and outcomes in the patients with ischemic CHF.

**Patients and Methods:**

This study was conducted on 154 patients with ischemic symptomatic moderate-to-severe CHF on discharge from hospital. The observation period was up to 3 years. Circulating NT-pro-BNP, TNF-alpha, sFas, and sFas ligand were determined at baseline. Flow cytometry analysis was used for quantifying the number of EMPs. All-cause mortality, CHF-related death, and CHD-re-hospitalization rate were examined. The data were analyzed using descriptive statistics, Receive Operation Characteristic Curve (ROC), and logistic regression analysis. Besides, P < 0.05 was considered as statistically significant.

**Results:**

During a median follow-up of 2.18 years, 21 participants died and 106 subjects were hospitalized repetitively. The results showed a significant difference between the patients with a large number of EMPs (> 0.514 n/mL) and those with a low level of the biomarker (< 0.514 n/mL) regarding their survival. The number of circulating EPMs independently predicted all-cause mortality (OR = 1.58; 95% CI = 1.20 – 1.88; P = 0.001), CHF-related death (OR = 1.22; 95% CI: 1.12 – 1.36; P < 0.001), and CHF-related re-hospitalization (OR = 1.20; 95% CI: 1.11 – 1.32; P < 0.001).

**Conclusions:**

Among the patients with symptoms of CHF, increased number of circulating EMPs was associated with increased 3-year CHF-related death, all-cause mortality, and risk of recurrent hospitalization due to CHF.

## 1. Background

Recent studies have suggested that endothelial dysfunction is crucial for clinical manifestations of Chronic Heart Failure (CHF) (1, 2). It has also been shown that endothelial monolayer injury leads to dramatic increase of endothelial-derived apoptotic microparticles (EMPs) (3). EMPs are a heterogeneous population of submicronic vesicles that are released in response to cell activation or apoptosis (4). EMPs represent an intercellular communication and delivery mechanism for efficient and effective transfer of biological information, leading to reprogramming of recipient cells, proatherogenic and prothrombotic effects, and modulating inflammatory response (5, 6). Increase in circulating EMPs is detectable in several cardiovascular diseases as well as in sepsis, cancer, chronic kidney disease, type two diabetes mellitus, and obesity (7-9). Although EMPs are considered as a marker of endothelial dysfunction and tissue remodelling (3, 10), their role as an indicator of inflammatory response and, probably, as a prognostic biomarker in CHF is still not clear.

## 2. Objectives

The present study aims to evaluate the relationship between EMPs and pro-inflammatory biomarkers, clinical status, and outcomes in the patients with ischemic CHF.

## 3. Patients and Methods

### 3.1. Patient Population

This study was conducted on 154 patients (86 males) aging 48 to 62 years old with ischemic symptomatic moderate-to-severe CHF. CHF was diagnosed according to the current clinical guidelines (11). Written informed consents for participation in the study were obtained from all the participants. The observation period was up to 3 years. Cumulative survival related to CHF as well as all-cause mortality was examined in this study.

### 3.2. Methods for Visualization of Coronary Arteries

Before enrollment of the patients into the study, their ischemic heart disease was verified through multispiral computed tomography angiography using Somatom Volum Zoom scanner (Siemens, Erlangen, Germany) with two detector rows at the end of breathing (12). After preliminary native scanning, non-ionic contrast Omnipaque (Amersham Health, Ireland) was administered for obtaining the optimal image of the coronary arteries.

### 3.3. Assessment of Hemodynamics

Left Ventricular (LV) end-diastolic and end-systolic volumes were measured by modified Simpson’s method (13) using ACUSON scanner (SIEMENS, Germany). Besides, tissue Doppler echocardiography was carried out in 4-, 3-, and 2-chamber projections in each of the 16 segments of the left ventricle and in 4 spots of the mitral annulus (14). In addition, peak systolic (Sm), early diastolic (Em), and late diastolic (Аm) myocardial velocities were measured in the mitral annulus area, followed by calculating the velocity of early diastolic LV filling (E) to Аm (Е/Аm) ratio and to Em (Е/Em) ratio.

### 3.4. Calculation of Glomerular Filtration Rate

Glomerular Filtration Rate (GFR) was calculated using MDRD-6 formula (15).

### 3.5. Measurement of NT-Pro-BNP, Total Cholesterol, and Its Fractions

Blood samples were taken in the morning (at 7 - 8 A.M.) and poured into cooled silicone test tubes. The samples were processed according to the recommendations of the manufacturer of the utilized analytical technique.

Circulating NT-pro-BNP level was measured by immunoelectro chemoluminescent assay using sets by R&D Systems (USA) on Elecsys 1010 analyzer (Roche, Mannheim, Germany). Moreover, the serum concentrations of TNF-alpha, sFas, and sFas ligand were determined in duplicate using commercially available enzyme-linked immunosorbent assay kits (Bender MedSystems GmbH, Vienna, Austria). Overall, 100 μL of the serum samples was assayed in parallel to determine the standard concentrations for each biological marker. The mean intra-assay coefficients of variation were < 10% of all the cases. The concentrations of Total Cholesterol (TC) and High-Density Lipoproteins (HDLP) were measured by fermentation method. On the other hand, Low-Density Lipoproteins (LDL-C) concentration was computed according to the Friedewald formula (1972). All the biomarkers were determined at baseline.

### 3.6. Assay of Circulating CD31 + /Annexin V + Endothelial-Derived Apoptotic Microparticles

EMPs were phenotyped through flow cytofluorimetry by phycoerythrin (PE)-conjugated monoclonal antibody against CD31 (BD Biosciences, USA) followed by incubation with fluorescein isothiocyanate (FITC)-conjugated annexin V (BD Biosciences, USA) using high-definition fluorescence activated cell sorter methodology. The samples were incubated in the dark at room temperature for 15 min according to the manufacturer’s instructions. The samples were then analyzed on a FC500 flow cytometer (Beckman Coulter) after 400 µL annexin-V binding buffer was added. Overall, 500 thousand events were analyzed for each sample. Furthermore, the EMPs gate was defined by size, using 0.8 and 1.1 mm beads (Sigma, St Louis, MO, USA). CD31 + /annexin V + microparticles were defined as EMPs positively labeled for CD31 and annexin V (CD31 + /annexin V +) (16).

### 3.7. Statistical Analysis

All the statistical analyses were performed using the SPSS statistical software, version 22 (SPSS Inc, Chicago, IL, USA). The data were presented as mean, Standard Deviation (± SD), 95% Confidence Interval (CI), median, and interquartile range. Student t-test or Shapiro–Wilk U-test were used to compare the main parameters of the patients’ groups. Additionally, Chi-square test and Fisher exact test were used to compare the categorical variables between the study groups. Moreover, the factors which could be potentially associated with circulating EMPs were determined by logistic regression analysis. Receive Operation Characteristic Curve (ROC) analysis was also performed to compare the cutoff points of the number of EMPs to the predicted values. Odds Ratio (OR) and 95% CI were calculated for all the independent predictors of patients’ survival. P < 0.05 was considered as statistically significant.

## 4. Results

### 4.1. General Characteristics of the Study Population

During a median follow-up of 2.18 years, 21 participants died and CHF-related death was detected in 18 patients. Additionally, 106 subjects were hospitalized repetitively due to advanced CHF (17 cases in the dead cohort and 89 ones in the survived cohort). According to Table 1, no significant difference was found between the patients who died and those who survived regarding age, sex, Body Mass Index (BMI), NYHA class, GFR, HbA1c, fasting blood glucose level, blood creatinine level, TC, LDL-C), HDL-C, and the number of damaged coronary vessels. The incidence of type 2 diabetes mellitus was 38.1% and 33.8% in the patients of the two cohorts, respectively (P = 0.060). Also, no significant difference was observed between the two cohorts concerning systemic office Blood Pressure (BP) and Heart Rate (HR). The results also showed no significant difference between the two cohorts regarding change in Е/Аm and Е/Em; however, decrease in the Left Ventricular Ejection Fraction (LVEF) was quite anticipated among the dead patients (Table 2). Besides, the level of circulating NT-pro-BNP was significantly higher in the dead patients compared to the survived ones. However, no significant difference was observed between the two cohorts with regard to administration of the majority of drugs.

**Table 1.** General Characteristic of the Study Patients

Variable	Died Subjects (n = 21)[Table-fn fn11847]	Survived Subjects (n = 133)
**Age, years**	57.20 ± 6.70	59.50 ± 7.30
**Males, n (%)**	12 (57.1%)	67 (50.3%)
**Arterial hypertension, n (%)**	12 (57.1%)	61 (45.9%)
**Dyslipidemia, n (%)**	9 (42.8%)	52 (39.1%)
**T2DM, n (%)**	8 (38.1%)	45 (33.8%)
**II NYHA Class**	6 (28.6%)	35 (26.3%)
**III NYHA Class**	9 (42.8%)	65 (48.9%)
**IV NYHA Class**	6 (28.6%)	33 (24.8%)
**BMI, kg/m** ^**2**^	23.7 (95% CI: 22.5 - 27.3)	24.2 (95% CI: 22.0 - 27.9)
**GFR, mL/min/1.73 m[Table-fn fn11848]** ^**2**^	82.1 (95% CI: 69.9 - 93.1)	85.2 (95% CI: 70.3 - 112.5)
**HbA1c, %**	6.3 (95% CI: 4.4 - 9.0)	7.0 (95% CI: 4.3 - 9.2)
**Fasting blood glucose, mmol/L**	4.80 (95% CI: 3.6 - 8.5)	5.40 (95% CI: 3.4 - 9.1)
**Creatinine, μmol/L**	70.5 (95% CI: 59.6 - 88.3)	74.9 (95% CI: 65.1 - 90.3)
**Total cholesterol, mmol/L**	5.3 (95% CI: 4.6 - 6.0)	5.0 (95% CI: 4.2 - 5.8)
**LDL-C, mmol/L**	3.60 (95% CI: 3.20 - 4.18)	3.02 (95% CI: 2.80 - 3.90)
**HDL-C, mmol/L**	0.94 (95% CI: 0.92 - 1.06)	0.88 (95% CI: 0.82 - 0.97)

Abbreviations: CI, confidence interval; CAD, coronary artery disease; T2DM, type two diabetes mellitus; GFR, Glomerular filtration rate; HDL-C, high-density lipoprotein cholesterol; LDL-C, Low-density lipoprotein cholesterol; BP, blood pressure; BMI, Body mass index; NYHA, New York Heart Association

^*^statistical differences between the two groups’ parameters (P < 0.05)

**Table 2.** Hemodynamic Performance, Natriuretic Peptide Level, Number of Coronary Artery Lesions, and Medications in the Study Patients

Variable Died	Subjects (n = 21)	Survived Subjects (n = 133)
**Systolic BP, mmHg**	129 ± 4	135 ± 5
**Heart rate, beats per 1 min**	76 ± 6	68 ± 3
**LVEF, %**	42.80 ± 0.76[Table-fn fn11849]	55.40 ± 0.80^*^
**Е/Аm, U**	16.6 ± 0.94	16.5 ± 1.20
**Е/Em, U**	16.6 ± 1.00	16.6 ± 0.84
**NT-pro-BNP, pg/mL**	1533.6 (95% CI: 644.5 – 2560.6)	1031.2 (95% CI: 704.8 – 1560.7)^ *^
**One-vessel lesion of coronary arteries, n (%)**	5 (23.8%)	24 (18.0%)
**Two-vessel lesion of coronary arteries, n (%)**	8 (38.1%)	54 (40.1%)
**Multi-vessel lesion of coronary arteries, n (%)**	8 (38.1%)	55 (41.4%)
**ACEI / ARAs, n (%)**	21 (100%)	133 (100%)
**Two-vessel lesion of coronary arteries, n (%)**	8 (38.1%)	54 (40.1%)
**Multi-vessel lesion of coronary arteries, n (%)**	8 (38.1%)	55 (41.4%)
**ACEI / ARAs, n (%)**	21 (100%)	133 (100%)
**Acetylsalicylic acid, n (%)**	19 (90.5%)	121 (91.0%)
**Other antiaggregants, n (%)**	2 (9.5%)	12 (9.0%)
**Statins, n (%)**	14 (66.7%)	80 (60.2%)
**Metformin, n (%)**	8 (38.1%)	45 (33.8%)
**Diuretics, n (%)**	18 (85.7%)	121 (91.0%)
**Mineralcorticoid receptors antagonists, n (%)**	9 (42.9%)	70 (52.6%)

Abbreviations: CI, confidence interval; BNP, brain natriuretic peptide; LVEF, Left ventricular ejection fraction; U, unit; Em, early diastolic myocardial velocity; Аm, late diastolic myocardial velocity; E, peak velocity of early diastolic left ventricular filling; ACEI, angiotensin-converting enzyme inhibitor; ARAs, angiotensin-2 receptors antagonists

^*^Statistical differences between the two groups’ parameters (P < 0.05)

### 4.2. Circulating EMPs, TNF-alpha, sFAS, and sFAS Ligand in Survived and Dead Patients

The median of EMPs circulating levels was 0.286 n/mL (95% CI, 0.271 - 0.309 n/mL) and 0.673 n/mL (95% CI, 0.65 - 0.74 n/mL) in survived and dead patients, respectively (P < 0.001). The concentrations of TNF-alpha, sFAS, and sFAS ligand were significantly lower in the survived patients compared to the dead ones (Table 3). In addition, the sFAS / sFAS ligand ratio was significantly higher in the dead cohort in comparison to the survived cohort (P < 0.001).

**Table 3.** Circulating Levels of EMPs, TNF-Alpha, sFAS, and sFAS Ligand in Both Cohorts

Variables	Survived Subjects		Died Subjects		P value
	Me	CI	Me	CI	
EMPs, n/mL	0.286	0.271 - 0.309	0.673	0.65 - 0.74 [Table-fn fn11851][Table-fn fn11852]	0.001
TNF-alpha, pg/mL	2.63	2.45 - 2.80	5.23	4.74 - 5.21	0.001
sFAS, ng/mL	2.96	2.75 - 3.17	5.17	4.90 - 5.47	0.001
sFASl, ng/mL	0.69	0.68 - 0.70	0.77	0.75 - 0.79	0.001
sFAS / sFASl ratio	4.22	3.96 - 4.49	6.7	6.37 - 7.02	0.001

Abbreviations: EMPs, endothelial-derived microparticles; TNF, tumor necrosis factor

Data presented as median (Me) and 95% CI

**Figure 1.**
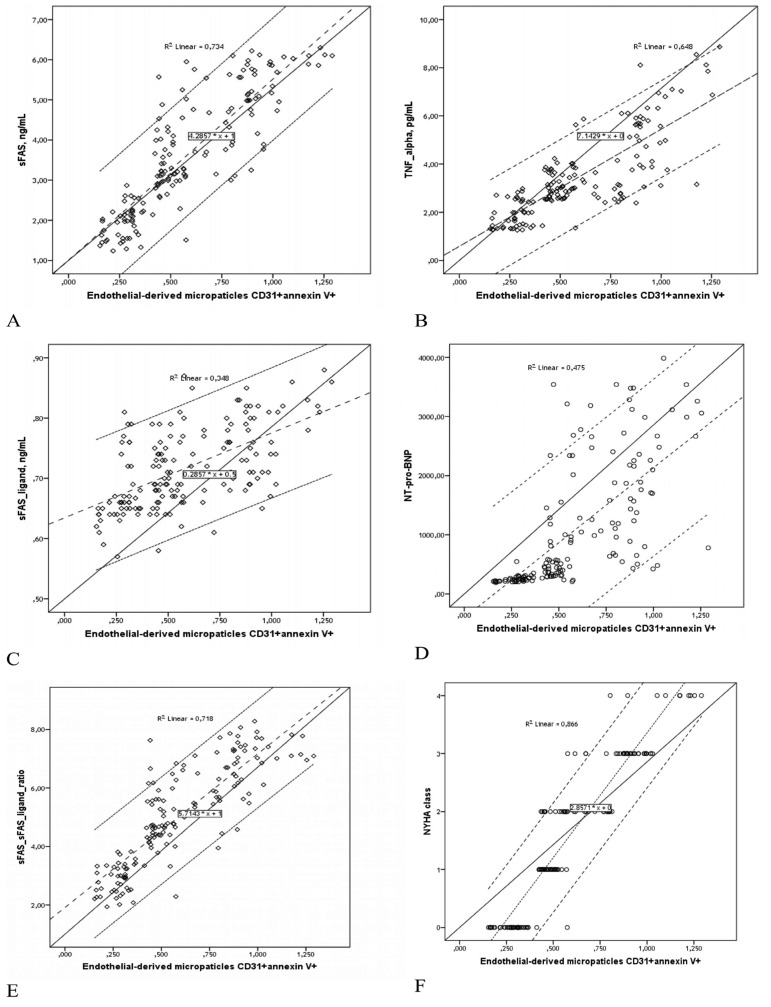
Scatterplots Show Association between EMPs Number in Plasma and sFAS (A), TNF-alpha (B), sFAS ligand (C), NT-pro-BNP (D), sFAS/sFAS Ligand Ratio (E), and NYHA Class (F) in Patient Population

The study results indicated a direct relationship between the number of EMPs in plasma and NYHA functional class (r = 0.93, P = 0.001), sFAS/sFASl ratio (r = 0.88, P = 0.001), sFAS (r = 0.856, P = 0.001), TNF-alpha (r = 0.827, P = 0.001), sFASl (r = 0.589, P = 0.001), and NT-pro-BNP (r = 0.689, P = 0.001). Also, a weak association was observed between the number of EMPs and type 2 diabetes mellitus (r = 0.402, P = 0.003), multi-vessel lesion of coronary arteries (r = 0.362, P = 0.001), Е/Аm (r = 0.360, P = 0.001), Е/Em (r = 0.344, P = 0.001), gender (r = 0.318, P < 0.001 for male), and TC (r = 0.313, P = 0.001). On the other hand, an inverse relationship was found between the number of EMPs and LVEF (r = -0.496, P = 0.001) and eGFR (r = -0.408, P = 0.003). Nevertheless, no significant association was observed between the levels of circulating EMPs and fasting plasma glucose, HbA1c, systolic and diastolic BP, premature CAD in family anamnesis, and medications in both cohorts (Figure 1).

#### 4.3. The Predictive Value of EMPs Number in the Study Population

**Figure 2.**
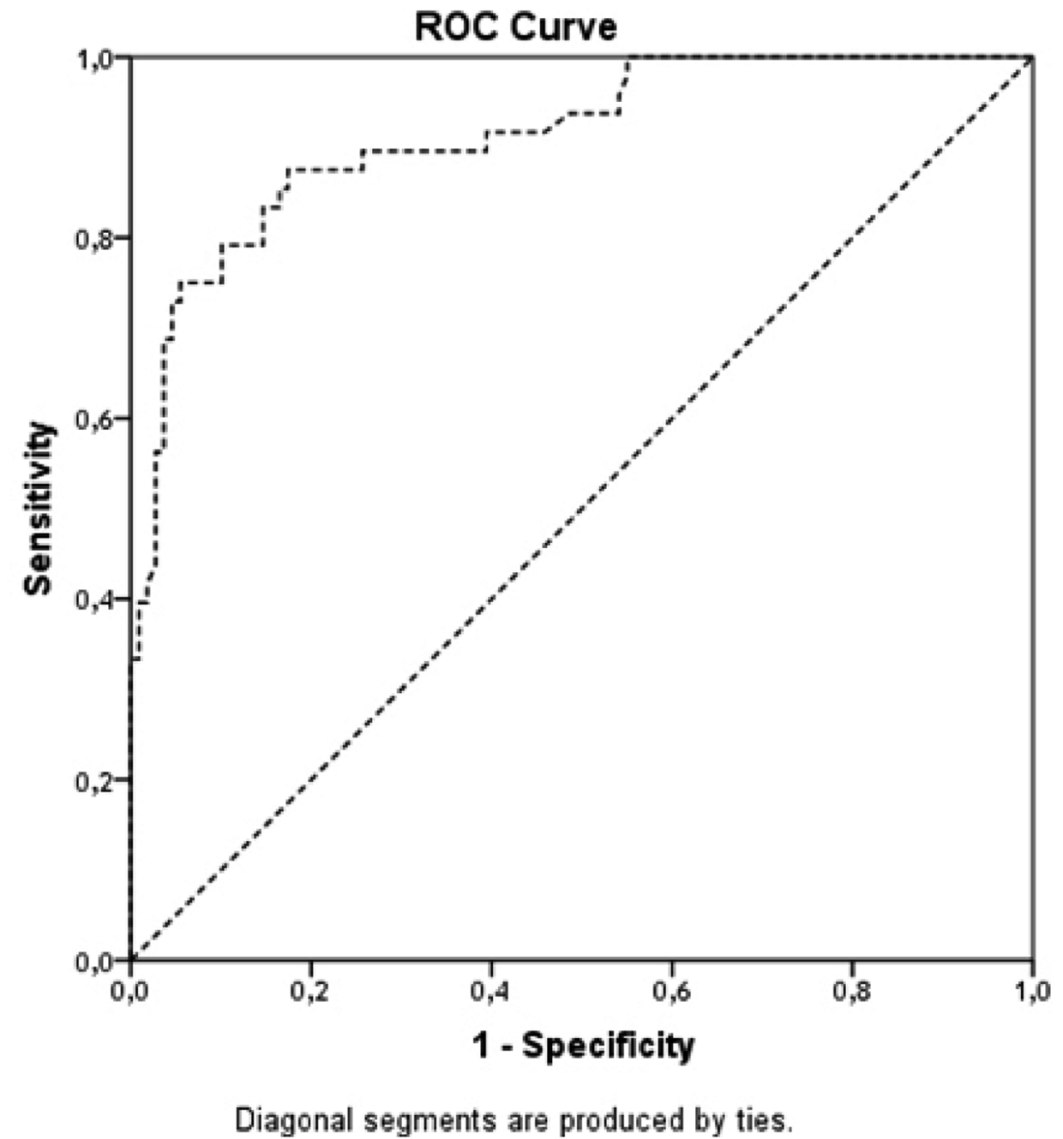
Reliability of the Model Included EMPs Number for Cumulative Survival in Study Patient Population; Results of the Receive Operation Characteristic Curve (ROC) Analysis. The Figure Shows the Ratio of Sensitivity and Specificity for Optimal Predict Number of EMPs.

**Figure 3.**
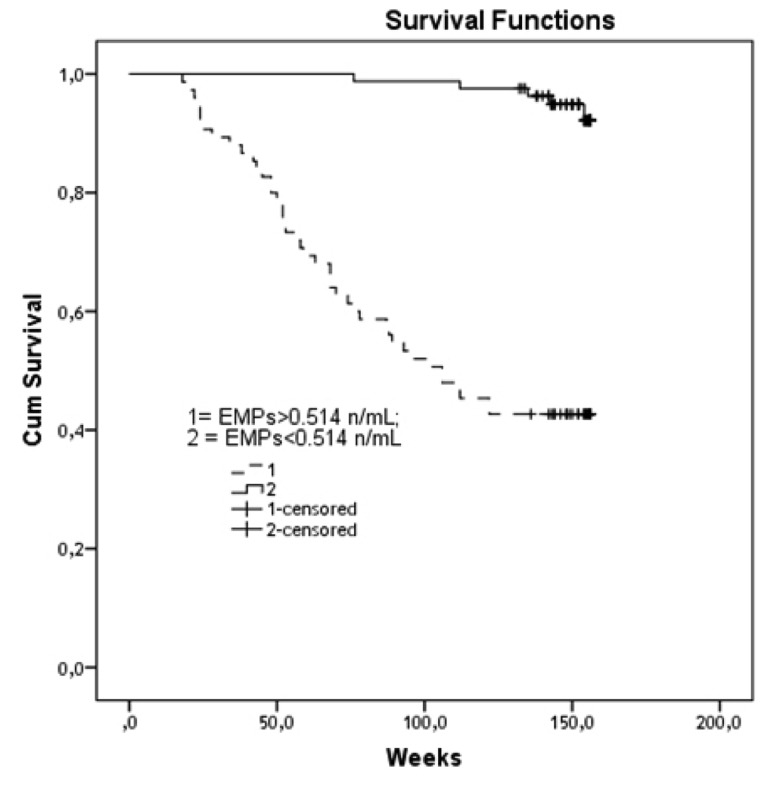
Distinguish of Survival Rate between Both Patient Cohorts with Low (< 0.514 n/mL) and High (> 0.514 n/mL) Numerous of EMPs; Results of Kaplan-Meier Survival Analysis

The optimum cut-off point for EMPs number in circulation was determined by the relative importance of the sensitivity and specificity of the test. ROC analysis showed that the cut-off point of EMPs number for cumulative survival function was 0.514 n/mL (Figure 2). Besides, the area under cure was 0.913 (Std. error = 0.025; 95% CI, 0.863 - 0.962) and the sensitivity and specificity of the test were 89.6% and 69.7%, respectively. The model was robust for all the occasions and provided significant results using the optimal cut-off point of EMPs (Table 4). The results revealed a significant difference between the patients with a large number of EMPs (> 0.514 n/mL) and those with a low level of the biomarker (< 0.514 n/mL) regarding their survival. The divergence of survival curves reached statistical significance in the 50th week of the observation period (P < 0.001) (Figure 3).

**Table 4.** The Association between Sensitivity and Specificity of EMPs Cut-off Point and Clinical Outcomes in the Study Population; Results of Receive Operation Characteristic (ROC) analysis

	Cut-off Point, n/mL	Sensitivity, %	Specificity, %	AUC (95% CI)	P value
**CHF-related death**	0.514	99.3	56.2	0.906 (0.843 - 0.970)	0.001
**CHF-related hospitalization**	0.514	87.5	65.0	0.86 (0.796 - 0.924)	0.001
**All-cause mortality**	0.514	99.6	57.4	0.906 (0.846 - 0.965) [Table-fn fn11853]	0.001

Abbreviations: AUC, area under curve; CI, confidence interval

Multivariate logistic regression was used to assess whether any combination of assays was able to better discriminate between the survived and dead patients. According to the results of logistic regression analysis, the main factors independently related to cumulative mortality and CHF-related rehospitalisations were EPMs, NT-pro-BNP, NYHA class, TNF-alpha, sFAS/sFAS ligand ratio, LVEF, type two diabetes mellitus, and three- and multi-vessel lesion. In addition, the number of circulating EPMs independently predicted all-cause mortality (OR = 1.58; 95% CI, 1.20 – 1.88; P = 0.001), CHF-related death (OR = 1.22; 95% CI, 1.12 – 1.36; P < 0.001), and CHF-related rehospitalization (OR = 1.20; 95% CI, 1.11 – 1.32; P < 0.001) (Table 5). NYHA class, NT-pro-BNP, TNF-alpha, sFAS/sFAS ligand ratio, and LVEF remained statistically significant for all the categories; i.e., all-cause mortality, CHF-related death, and CHF-related rehospitalizations, whereas type two diabetes mellitus and three- and multi-vessel lesion did not.

**Table 5.** Variables Independently Related to 3-Year All-Cause Mortality, CHF-Related Death, and CHF-Related Rehospitalisation, Obtained by Logistic Regression Analysis

Variables	All-Cause Mortality			CHF-Related Death			CHF-Related Rehospitalisation		
	OR	95% CI	P	OR	95% CI	P	OR	95% CI	P
**EMPs**	1.58	1.20 – 1.88	0.001	1.22	1.12 – 1.36	0.001	1.20	1.11 – 1.32[Table-fn fn11854]	0.001
**NYHA class**	1.12	1.01 – 1.24	0.05	1.18	1.05 – 1.30	0.001	1.12	1.07 – 1.22	0.001
**TNF-alpha**	1.10	1.03 – 1.17	0.016	1.12	1.02 – 1.19	0.012	1.16	1.10 – 1.26	0.001
**sFAS / sFAS ligand ratio**	1.12	1.06 – 1.20	0.001	1.14	1.08 – 1.22	0.001	1.15	1.12 – 1.19	0.001
**NT-pro-BNP**	1.09	1.02 – 1.16	0.002	1.42	1.22 – 1.73	0.006	1.44	1.28 – 1.67	0.002
**LVEF**	1.06	1.01 – 1.12	0.001	1.15	1.12 – 1.18	0.014	1.22	1.07 – 1.45	0.016
**T2DM**	1.05	1.01 – 1.11	0.001	1.03	0.93 – 1.10	0.32	1.04	0.97 – 1.06	0.42
**Three- and multi-vessel lesion**	1.02	0.88 – 1.09	0.56	1.01	0.92 – 1.07	0.27	1.14	1.03 – 1.26	0.012

Abbreviations: OR, odds ratio; CI, confidence interval; LVEF, left ventricular ejection fraction; BNP, brain natriuretic peptide; T2DM, type two diabetes mellitus

Using a stepwise model selection method for multivariable prediction model, we investigated the summary effect of any combinations of EMPs, NT-pro-BNP, TNF-alpha, sFAS/sFAS ligand ratio, and LVEF on all-cause mortality, CHF-related death, and CHF-related rehospitalizations (Table 6). According to the results, EMPs number alone (Model 1), combination of EMPs number and NT-pro-BNP (Model 2), combination of EMPs number and NT-pro-BNP and TNF- alpha (Model 3), and combination of EPMs and NT-pro-BNP + NYHA class + TNF-alpha (Model 4) were statistically significant predictors of all-cause mortality, CHF-related death, and CHF-related rehospitalizations, whereas Model 5 (EPMs + NT-pro-BNP + NYHA class + TNF-alpha + sFAS/sFAS ligand ratio) and Model 6 (EPMs + NT-pro-BNP + NYHA class + TNF- alpha + sFAS/sFAS ligand ratio + LVEF) were not. The stepwise model selection method demonstrated that NYHA class, NT-pro-BNP, LVEF, TNF-alpha, and sFAS/sFAS ligand ratio added to EMPs did not offer any additional information to discriminate between the survived and dead patients with symptomatic ischemic CHF (B-coefficient = 0.14, 0.018, 0.086, 0.092, and 0.016, respectively; P values = 0.86, 0.65, 0.58, 0.54, and 0.56, respectively).

**Table 6.** Predictive Value of EPMs/MPCs Ratio for Combined End-Point (All-Cause Mortality and CHF-Related Re-Hospitalisations); Multivariable Prediction Model Showed Lack of Additional Information to Discriminate between Survived and Dead Patients with Symptomatic Ischemic CHF When NT-pro-BNP, NYHA Class, LVEF, Type Two Diabetes Mellitus, and Three- and Multi-Vessel Lesion of Coronary Arteries Were Added to EPMs/MPCs Ratio.

	OR	95% CI	P value
**Model 1** ^**a**^	1.62	1.12 – 1.92	0.001
**Model 2** ^**b**^	1.56	1.09 – 1.77	0.003
**Model 3** ^**c**^	1.42	1.12 – 1.61	0.001
**Model 4** ^**d**^	1.25	1.07 – 1.62	0.003
**Model 5** ^**e**^	1.01	0.88 – 1.07	0.02
**Model 6** ^**f**^	0.92	0.91 – 1.109	0.22

^a^ Model 1: EPMs;

^b^ Model 2: EPMs + NT-pro-BNP;

^c^ Model 3: EPMs + NT-pro-BNP + NYHA class;

^d^ Model 4: EPMs + NT-pro-BNP + NYHA class + TNF-alpha;

^e^ Model 5: EPMs + NT-pro-BNP + NYHA class + TNF-alpha + sFAS / sFAS ligand ratio;

^f^ Model 6: EPMs + NT-pro-BNP + NYHA class + TNF-alpha + sFAS / sFAS ligand ratio + LVEF

## 5. Discussion

Circulating EMPs play a pro-inflammatory detrimental role in the vascular dysfunction that is a key mechanism in development and progression of a wide range of cardiovascular diseases (17). EMPs may trigger endothelial dysfunction by disrupting the nitric oxide release from vascular endothelial cells (3, 8). Circulating EMPs affect both pro-inflammatory and pro-atherosclerotic processes, promote coagulation and inflammation, and modulate angiogenesis and apoptosis in endothelial cells (18-20). The Fas ligand (FasL) induces apoptosis or programmed cell death when bound to its membrane receptor Fas. FasL is able to impair the survival of endothelial cells (21). A recent study has revealed an association between circulating EMPs labelled as CD31+ / annexin V+ cells and cardiovascular outcomes (10). In our investigation, a significant increase of EMPs level in circulation was detected in the ischemic CHF patients who had died compared to those who had survived. Because endothelial cells express FasL constitutively and TNF-alpha contributes to apoptosis through affecting Fas-Fas ligand system (22), we suggested that increase of the EMPs levels in the CHF patients might be associated with TNF-alpha and FAS-sFAS ligand ratio. Indeed, the results indicated a correlation between TNF-alpha, FAS, sFAS ligand, and FAS-sFAS ligand ratio and EMPs level. Also, a close relationship was observed between EMPs level and NT-pro-BNP as well as NYHA class. Thus, we confirmed that pro-inflammatory activation was dramatically increased in the CHF subjects who were at a high risk of death. Moreover, the associates between endothelial dysfunction and EMPs level might reflect the severity of endothelial dysfunction due to systemic inflammation In this sense, the CHF patients with low levels of EMPs (< 0.514 n/mL) demonstrated a superior survival compared to those with high levels of EMPs (> 0.514 n/mL). Furthermore, multivariable prediction model showed the highly decremented potential of EMPs alone in CHF patients. These findings suggest that increased EMPs number might improve the predictive value of contemporary model in CHF based on clinical performance and NT-pro- BNP measurement. Although the cellular mechanism of action of EMPs largely remains unclear, increase in the number of EMPs in CHF might reflect a reduced vascular repair capacity and severity of endothelial dysfunction that is probably considered as staging disease. In this study, levels of EMPs and NT-pro-BNP independently predicted long-term cumulative survival, rehospitalization due to CHF, and CHF-related death. Yet, it should be mentioned that although EMPs have a large diagnostic potential as biomarkers in cardiovascular diseases and cancer, due to the current technological limitations in purification of EMPs and an absence of standardized methods of detection, the role of EMPs has become controversial (23). Hence, further studies with higher statistical power are needed to assess whether EMPs levels constitute a prognostic marker in CHF. Moreover, knowledge of the functional properties of EMPs will contribute to a better understanding of the pathological mechanisms of communication between pro-inflammatory activation and CHF progression, because EMPs may be an attractive prognostic biomarker for CHF.

Among the patients with symptoms of CHF, increased number of circulating EMPs was associated with increased 3-year CHF-related death, all-cause mortality, and risk of recurrent hospitalization due to CHF. This also provides an additional explanation by which low-intensity inflammation that is suitable for CHF may contribute to development of endothelial dysfunction and might reflect a risk of unfavorable clinical outcomes irrespective of traditional biomarkers, such as NT-pro-BNP, NYHA class, and LVEF.

### 5.1. Ethical Principles

The study was approved by the local Ethics Committee of State Medical University, Zaporozhye, Ukraine. The study was carried out in conformity with the Declaration of Helsinki.

### 5.2. Study Limitations

This study had some limitations. The authors believe that further studies should be conducted on a larger cohort of patients to improve the power of the study. Of course, these restrictions might have had no significant impact on the interpretation of the study results.
